# Distributed Simulation System for Athletes' Mental Health in the Internet of Things Environment

**DOI:** 10.1155/2022/9186656

**Published:** 2022-03-25

**Authors:** Baoyan Fu, XinXin Fu

**Affiliations:** Jiangxi Teachers College, Yingtan, Jiangxi 335000, China

## Abstract

Psychological troubles in training competitions mainly include worry about mistakes, long-term lack of improvement in sports performance, and lack of confidence in competitions. The main troubles in daily study and life are future career development and life planning, injury and illness, insomnia, and poor emotional control. Athletes are interested in psychological skills training, hobby training, interpersonal communication and other coaching content to improve sports performance. Athletes tend to prefer one-to-one psychological counseling and group counseling activities; there are differences in the psychological distress, coping styles and expected psychological counseling content of athletes in different age groups and events. This paper firstly introduces the important role of psychological quality education in modern competitive sports. The influencing factors of athletes' psychological quality were analyzed. At the same time, combined with relevant practical experience, it starts from various perspectives and aspects such as improving the scientific literacy of coaches and building a harmonious atmosphere for training and competition. This paper puts forward some effective strategies to strengthen athletes' psychological quality education and improve sports performance. In addition, it expounds the author's understanding of this, hoping to contribute to the practice of athletes' psychological quality education.

## 1. Introduction

**Figure 1:**
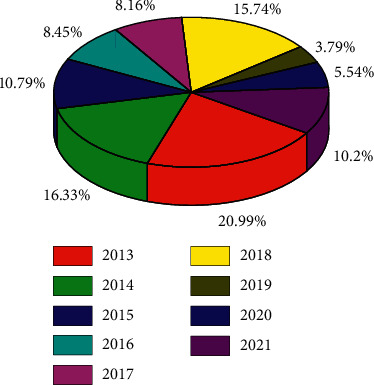
The occurrence of mental problems in athletes over the years.

In modern competitive sports, the intensity and difficulty of competition are getting bigger and bigger, which puts higher demands on the physical and mental health of athletes. Under the current situation, we must pay attention to the important role of psychological quality education in the application of modern sports. Objectively examine the many influencing factors affecting the mental health of competitive athletes, grasp the key points of the mental quality education of athletes, and promote the optimization and improvement of sports performance [1]. Based on the current situation of the needs and deficiencies of health monitoring and management of various types of athletes, athletes need to maintain a good mental state during training and competition. This is because a good mental state is conducive to its maximum potential and bear greater pressure. Generally speaking, they can be divided into three categories. The first category, such as smart bracelets or watches, has the function of automatic health data collection, but lacks follow-up analysis and connection with health services. The second category, such as online health analysis and diagnosis platforms, can generate health reports based on big data, but data collection is not automatically connected to no health services. The third category, such as fitness or nutrition recommendation apps, passively provides health services, such as fitness courses, but is not based on intelligent recommendation and drainage based on data analysis [2]. Therefore, there is an urgent need for a digital platform that integrates real-time collection and monitoring of personal health data, automatic generation of health analysis reports, and follow-up intelligent recommendation to connect with health services. It is based on the above-mentioned status quo, to carry out research on the application of the Internet of Things and artificial intelligence to realize a health monitoring and management platform that spans time and space [3]. Based on the above analysis of the status quo and deficiencies, there is currently no platform or product that connects health data detection and subsequent health recovery services. Therefore, the *IoT* cutting-edge technology that integrates the Internet of Things, artificial intelligence deep learning and intelligent recommendation algorithms is proposed to realize the three-in-one process of “data based health monitoring and management service platform research based on the Internet of Things and deep learning” [4]. Research on health monitoring management service platform is significant for maintenance of athletes' health. When athletes use the platform, they can achieve accurate collection of health data and real-time update and view of health data through *IoT* collection devices. In addition, it will also recommend a series of health service management such as one-to-one live teaching by fitness coaches, one-to-one consultation with professional nutritionists, tweeting services on health forums, and Internet + diagnosis and treatment services based on the athletes' current physical health data. The health monitoring and management service platform based on the Internet of Things and deep learning can serve everyone who cares about their own health, and is precisely positioned for athletes with fierce competition and difficult psychological construction. Automatic real-time health data detection of users through IoT devices, big data analysis, and intelligent generation of user health monitoring analysis reports [5]. As shown in Figure 1, they are also students with rapid physical and mental development, and they will also face the problems that ordinary teenagers will encounter in their growth, such as academic pressure and internal contradictions in physical and mental development. The two influence each other and have their own particularities. In the previous International Olympic Committee, experts also emphasized that while paying attention to the psychological quality of athletes' competitive performance, it is necessary to pay attention to the mental health problems that often occur in athletes [6–9].

The full use of information technologies such as the Internet of Things in athletes' service management can effectively improve the technical quality and safety assurance of athletes' mental health management services. This approach can effectively enhance the high importance of effective management of their own health for all types of athletes. While effectively reducing the cost of psychological medical expenses, it can also change the bad mental life and behavior of athletes, and can effectively prevent the occurrence of mental diseases in athletes. In this way, it can effectively promote the recovery of athletes after various diseases, and further improve the economic, material and spiritual life of college teachers and students [10]. The Internet of Things project includes infrared sensors, laser scanners, and information sensors. According to the relevant protocol requirements, the connection between items and the Internet is realized, and the information flow between them is realized, so as to achieve the purpose of monitoring, tracking, identification, positioning and management. With the gradual increase in the number of sports and competitions in our country, there are higher requirements for venues, security and athlete training facilities. With the development of the Internet of Things project, it has brought more interactivity to the development of sports in our country, and has a very good development situation. It can be said that the development of the Internet of Things project. It will definitely bring significant changes to the development of sports in our country [11]. Athletes' mental health issues are getting more and more attention. Previous researchers have generally used tools such as clinical screening tools or mood state scales from a 90-item symptom list to assess the mental health of athletes. Although these tools are widely used and have shown good reliability and validity in many groups, their use in the field of sports has exposed the difficulty of daily monitoring and screening of sports teams and the irrelevance of the content of the scale to the sports context. Rice developed the Athlete's Psychological Stress Questionnaire from the perspective of the most obvious psychological stress on the development of athletes' mental health symptoms for daily monitoring and screening of professional athletes' mental health status [12]. Psychological stress refers to a state of feeling stressed and difficult to cope with, and is a state of constant emotional exhaustion and reaction to stressful experiences. Compared to internalized symptoms that other clinical diagnostic tools focus on, psychological stress can screen athletes for early signs of possible mental health problems for early professional support [13, 14]. In recent years, there have been more and more mental health problems among adolescents, which is worrying and has attracted attention from all walks of life. The relevant functional departments of the state, provinces and cities have also issued documents on the mental health of young athletes for many times, and the research on the mental health of young athletes is also in-depth. However, there are relatively few studies on the mental health of young athletes. Based on this, this study starts from the Internet of Things technology to investigate the mental health of athletes in youth sports training centers, establishes a health analysis system model. The real-time stream of data from IoT sensors, combined with historical data from other projects, can be used to monitor the current work site and provide an ever-increasing set of data that can be used for simulating system to monitor athletes' mental health. This study aims to investigate the mental health status of athletes based on the Internet of things technology, establish a health analysis system model, and compare the effects of different factors on Athletes' mental health by horizontal and vertical simulation.

## 2. A Brief Introduction to the Internet of Things

The concept of the Internet of Things was first proposed by the *MIT Auto-ID* Research Center in 1999. This technology is a technology that can use radio frequency identification technology to identify and manage the researched affairs and laws anytime and anywhere under network conditions, which is what we call the interconnection between items. The essence of the technology is mainly to effectively integrate radio frequency identification (*RFID*) technology and the Internet, and to effectively apply the integrated products. *RFID* is a wireless communication technology that can use radio signals to identify a specific target and read and write relevant data without the need to establish mechanical or optical contact between the identification system and a specific target.

**Figure 2:**
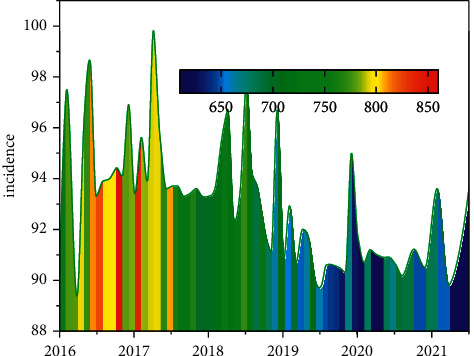
Percentage of athletes with mental problems over the years.

As shown in Figure 2, the emergence of the Internet of Things can effectively solve the interconnection between objects to objects, people to people, and people to objects. The Internet of Things is also quite different from the traditional Internet. For example, the interconnection between people and objects we are talking about mainly refers to the connection between people and objects through the use of some common devices. The interconnection between people mainly means that the process of interconnection between people no longer only depends on the connection method of personal computers [15].

(1) $$\begin{array}{c} E_{k}=0.5\sum_{i=1}^{q}{\left(Y_{i}^{k}-C_{t}^{k}\right)}^{2}. \end{array}$$

In the formula, *E*_k_ represents the error between the expected value and the actual value of the neural network.

The application of the Internet is based on optical fiber, to form a network of multiple computers to realize the communication and sharing of information. However, the network environment established by the Internet is actually virtual, and people can only apply the virtual information existing in it, but cannot change the form of real objects in the world. The emergence of *IoT* technology is to remedy this problem. At the same time, the Internet of Things can also realize the unmanned driving of vehicles, so that they can automatically avoid vehicles and congested roads. Thereby avoiding the occurrence of other situations such as traffic jams and saving more time for people. Based on this, we can find that the application of Internet of things engineering has high requirements for relevant equipment and devices. It needs a certain effectiveness and stability to improve people's work efficiency and quality of life [16].

**Figure 3:**
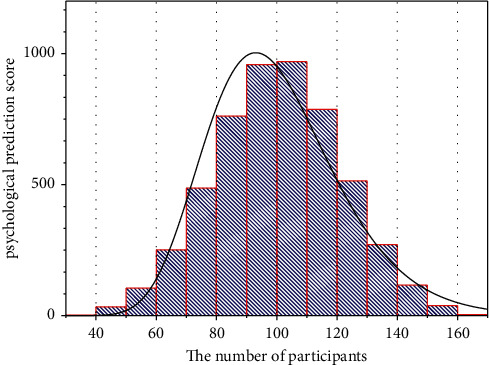
Psychological evaluation score curve of participating athletes.

The Internet of Things realizes the Internet between objects, through sensing devices, *RFID* technology, and identification technology. As shown in Figure 3, according to relevant protocols, the network connection between objects is realized, and the information exchange between objects is realized, so as to achieve the purpose of intelligent tracking, positioning, management and monitoring. This intelligent system will be combined with media, services and enterprises in the future to form the future Internet model [17]. The exact methods of data processing based on the SPSS software are data trend analysis, data contrast analysis and data subdivision analysis.

The essence of the Internet of Things is a convergent application in the development of information technology, and its characteristics mainly include the following aspects. First of all, the Internet of Things has the characteristics of the Internet, and it needs to be connected to the Internet before the intercommunication between objects can be realized. Secondly, a supervision cloud platform for environmental protection functional departments is established. Finally, the Internet of Things must be intelligent. The intelligence mentioned here refers to self-feedback ability, intelligent control ability and automatic application ability. According to the above description, the application of the Internet of Things project in the mental health of athletes will greatly promote the development of sports in my country. In sports, it is necessary to bring out the potential of people's physical, psychological, athletic ability and physique, so it is necessary to formulate a series of training programs and methods [18].

(2) $$\begin{array}{c} F_{i}=\frac{\left(x_{i}-\bar{x}\right)}{\bar{x}}\times 100\%. \end{array}$$

In the formula, *F*_*i*_ represents the deviation of the index from the mean value of the index group. The larger the value, the higher the mean value, and the lower the mean value on the contrary.

**Figure 4:**
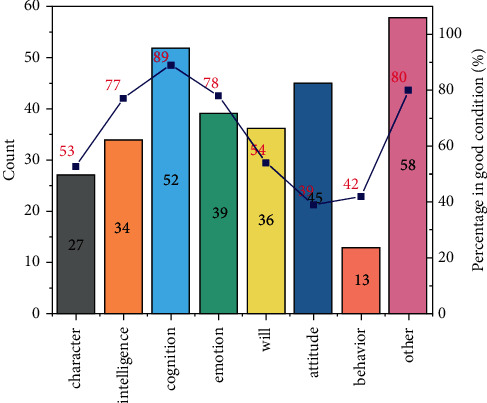
Psychological and behavioral impact levels.

At present, most of the health management work in my country is carried out by physical examination institutions and medical institutions, but in actual work, their focus is disease control rather than health management in the true sense. As shown in Figure 4, with the popularization of the concept of the Internet of Things, some people are now focusing on monitoring various indicators of the human body in real time through the use of computer and mobile phone software. As a result, the factors threatening the mental health of athletes are simulated and analyzed in the early stage, and effective solutions are proposed. Make a huge contribution to the development of the sports industry. The Internet of Things has become a game changer for all sports. The IoT sensors are now being used to measure minute details of all aspects of performance to help athletes improve their performance, such as running, swimming, cycling and so on [19, 20].

## 3. Operation of Mental Health Management Based on Internet of Things

According to the market survey and relevant statistical results of relevant research institutions in our country, it is known that in recent years, the mental and physical quality of various athletes in many countries around the world needs to be further improved. This is because with the rapid progress of the people, the competitiveness in sports competitions is also increasing. Correspondingly, it will also bring huge psychological pressure to many athletes, so many relevant institutions and organizations have made great efforts to increase the importance and degree of education on athletes' mental health literacy. Mental health test management needs to include a lot of test content, among which athletes' mental health management test and physical fitness evaluation management are very critical parts. Therefore, to seriously and effectively improve athletes' physical and psychological quality, we must work hard. Create a rational model for the management of mental health testing of athletes [21, 22]. The continuous real-time stream of data from IoT sensors, combined with historical data from other projects, can not only be used to monitor the current work site. However, it also provides a set of data that can be used for machine learning for predictive analysis to make athletes' mental health monitoring system smarter.

### 3.1. Correct Choice of Frequency

**Figure 5:**
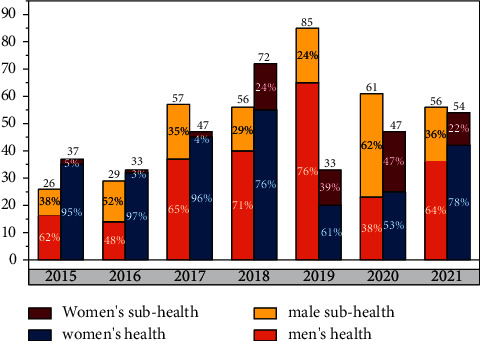
Health status of male and female athletes over the years.

According to the performance classification and characteristics of its operating frequency, electronic tags can be divided into low frequency (*LF*: 30–300 kHz), high frequency (*HF*: 3–30 MHz), ultra-high frequency (*UHF*: 300MHz-3 GHz) and microwave frequency band (*MW*: 2.45–5.8 GHz) four frequency bands. *RFID* applications in the international are generally based on *LF* and *HF* tags as the main implementation. As shown in Figure 5, at this stage, the relevant institutions has mastered the core design technology of *HF* chips, and has already put it into actual mass production applications on a large scale. The data in Figure 5 comes from public data resources published on the Internet. Meanwhile, the *UHF* chip has also successfully completed the research and development process and development stage [23].

### 3.2. Privacy and Security Protection Measures

*RFID* product labels have their own unique characteristics, but in fact they should all have a unified product code based on the global electronic product label code standard or a unique product code in the world. And this product label has been written into a corresponding product label before their packaging work is completed or before they leave the factory, and it is not allowed to read and write settings at will. At the same time, all the databases in the core chip are fully encrypted in parallel and have passed international security certification indicators, which can fully ensure the security of data sources and processed information. This can not only effectively prevent the artificial cracking of encrypted links and other databases, but also automatically lock some of our important confidential data information according to the sector mechanism, further ensuring that our data security will not be leaked.

(3) $$\begin{array}{c} \Delta v_{j}=-\alpha \left(\frac{\partial E_{k}}{\partial v_{j}}\right)=\alpha d_{t}^{k}b_{j}. \end{array}$$

In the formula, the correction value of the network weight is Δ*v*_*j*_, which represents the influence strength of the neural network error change on the output result.

### 3.3. System Cost Analysis

In the context of the social Internet, the cost of electronic tags has become lower and lower. Since 2006, the price of each tag has dropped to 5 cents. At the same time, because the tag can realize the functions of reading and writing data, and can also be recycled many times, the cost of the tag becomes lower and lower.

(4) $$\begin{array}{c} \Delta w_{ij}=-\beta \left(\frac{\partial E_{k}}{\partial w_{ij}}\right)=\beta E_{j}\alpha_{i}. \end{array}$$

In the formula, △*w*_*ij*_ represents the change value of the connection weight from the hidden layer to the input layer.

### 3.4. International Standard Issues

At present, the 2009 edition of *RFID* and other related information technology and electronic equipment development and application standard specifications and related data information of the standard are being researched and formulated internationally. These sources mainly use the International Organization for Standardization (*ISO/IEC*), and currently the main standards for my country are all based on the 2009 edition of *ISO/IEC* 15693. Basically, the specific research or drafting of the industry technical standards related to the national standards of the industry has been successfully completed. With reference to the relevant national standards formulated by *ISO/IEC*1800 in 2009, their standards have also been officially included in the industry standard national key project implementation plan.

(5) $$\begin{array}{c} \Delta \theta_{j}=-\beta \left(\frac{\partial E_{k}}{\partial \theta_{ij}}\right)=\beta E_{j}. \end{array}$$

In the formula, *θ* represents the threshold change of the hidden layer.

### 3.5. Implementation of System Design Plan

**Figure 6:**
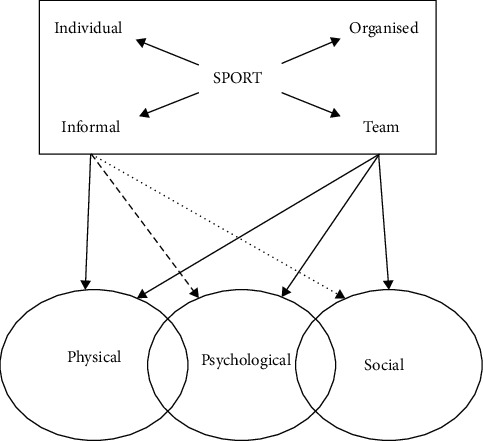
The relationship between mental and social health in sports.

Take advantage of some current hospital management systems and professional local area networks. Such as the *HIS* and *LIS* of large universities, institutions and government public administration departments, and on this basis initially complete the treatment of hospital patients, equipment and medical equipment. The system is mainly used in clinical medicine, constitutes the main part of the system, and is the system hardware and related software for optimizing the management of commonly used drugs. We can see from Figure 6 that the most important parts of the hardware include *RFID* network tags, *RFID* network antennas, file readers, *RFID* operating systems, servers, terminals, and related network cables. And each component is mostly connected in larger shapes by different network devices, all of which are equivalent to a broad network running an operating system. However, software functionality generally refers to the software applied to all *RFID* tag servers and tag users for each mobile station.

**Figure 7:**
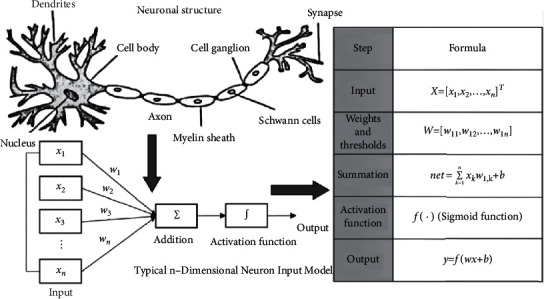
System structure diagram.

Through the use of the Internet of Things method, the real-time management of sports equipment can be realized, thereby helping athletes to reasonably allocate time and methods during exercise. We can see from Figure 7 that real-time monitoring of the warehousing and delivery of sports equipment, record the application of equipment and equipment, and share the records to improve the application efficiency of sports equipment. In this way, it can effectively improve the training efficiency of athletes and greatly reduce their psychological burden.

(6) $$\begin{array}{c} p\left(y|x\right)=\frac{e^{yx^{T}w}}{1+e^{yx^{T}w}}. \end{array}$$

This formula represents the mathematical definition of logical distribution under this model, which is a method to complete the classification based on the discriminant of logical distribution.

## 4. Application of Internet of Things Engineering in Sports

**Figure 8:**
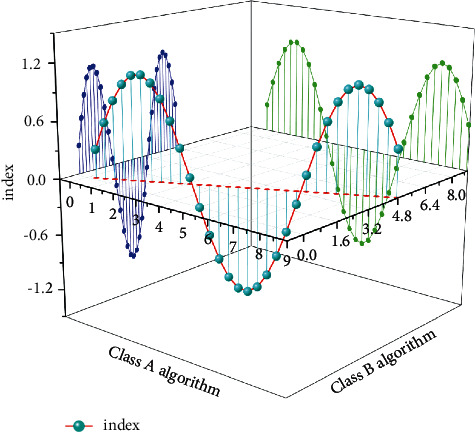
Comparison of the two algorithms.

Using the Internet of Things project to monitor sports is actually monitoring the physical conditions of athletes in real time, because these physical conditions can reflect psychological changes to a certain extent. The terminal data collection method is used for information analysis. Once abnormality is found in the relevant parameters, an alarm will be issued immediately, thereby reminding the coaches and team doctors to take corresponding psychological counseling to avoid the occurrence of sports accidents during training. We can see from Figure 8 that this monitoring method can be applied to sports training as well as competitive competitions. Athletes can use the Internet of Things to monitor their physiological parameters during the usual training process. For example, for sprinters, they need to have strong explosive power, and in the process of sprinting, the heart will instantly enter. In the state of high-speed operation, how to train to improve the ability of the heart is very important.

(7) $$\begin{array}{c} f\left(w;X,y\right)=L\left(w;X,y\right)+R\left(w\right). \end{array}$$

The above formula represents a model that rewrites the objective function in the form of a loss function.

Compared with training, athletes will continue to be in a state of high-intensity exercise in competitive competitions, and we can sometimes see athletes die suddenly during competitions. So far, hundreds of athletes have died suddenly during the competition due to too intense exercise. A very important reason for this phenomenon is that athletes do not pay attention to psychological adjustment during high-intensity training. If real-time monitoring of organs in athletes can be carried out, athletes can be warned before organ problems occur. Thereby, it can stop exercising, and effectively improve the psychological counseling, thereby avoiding the phenomenon of excessive exercise of athletes. Athletes cannot directly perceive their own mental state, so IoT engineering is needed to provide technical support.

(8) $$\begin{array}{c} c_{j}^{*}=\frac{1}{n_{j}}\sum_{i=1}^{n_{j}}x_{i}^{j}. \end{array}$$

In the formula, the number of mental health data objects in the *c*_*j*_ cluster is *n*_j_.

As the continuous development of Internet cloud computing technology and various hardware facilities, some equipment systems using the Internet of Things cloud technology have emerged. This is a health management system that includes various subsystems such as various biological detection instrument systems, wireless signal transmission and reception systems, terminal data processing systems, and background database storage and processing systems. In S mode, the data on the server can be added, modified and deleted through their own terminal machine, and the analysis results and exercise intervention plan fed back to the terminal by the background server can also be obtained. Users can exercise according to the plan. The data during the exercise will be transmitted to the background server to further guide psychological counseling.

(9) $$\begin{array}{c} J^{*}={\sum_{k=1}^{n_{j}}\sum_{j=1}^{k}\left\|x_{k}^{j}-c_{j}^{*}\right\|}^{2}. \end{array}$$

If |*J*^*∗*^ − *J*| < *ξ*, it means that the clustering criterion function converges, and the final clustering result is obtained, and *ξ* is the criterion for judging the convergence of the function.

The mental health monitoring work process of athletes generally generates a huge amount of data, and the analysis of these health monitoring data has extremely important practical significance. More work mistakes make the mental health monitoring work of the athletes lose its value.

(10) $$\begin{array}{c} A_{i}=\frac{\sum_{i=1}^{3}a_{y}\partial_{y}}{\sum_{i=1}^{3}y}. \end{array}$$

In the formula, *A*_j_ is the value corresponding to each feature layer index calculated, and the coefficient of each base layer is determined, and the value is 0–1.

The popular science service provided by the athlete mental health tracking system is no longer the original one-way indoctrination, but it is changing to two-way interaction, that is, the two-way communication between the disseminator and the recipient of the popular science service is realized by using the product of the athlete mental health tracking system as the communication medium. In addition, while the athlete mental health tracking system provides popular science and services to all sports circles, these health data can become the basis for the investigation of athletes' mental health quality. The athlete mental health tracking system is a service-oriented popular science system, which provides a good opportunity for the development of future popular science exhibits. The design of this system fully reflects the people-oriented concept, and the design is more in line with the actual needs of athletes advantages of the Internet.

## 5. The Important Role of Psychological Quality Education in Sports Competition

With the development of economy and society, sports competition has been continuously improved and has become a key part of human social life. In the complex, ever-changing and exciting competitive arena, only with stable and strong psychological quality can we better play our due level and achieve better sports results. In sports, the contest between athletes is not only a contest of their own technical level, but also a contest of psychological quality. When athletes of the same level compete on the same field, the ups and downs of psychological state become the decisive factor for the performance of athletes.

**Figure 9:**
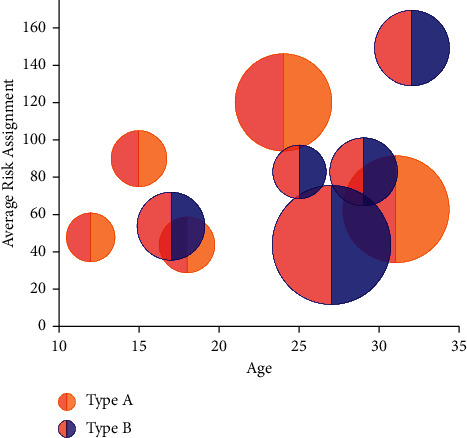
Average risk assignment for different ages.

Mental health is a key component of a person's overall quality. The level of modern competitive technology has generally improved, and the audience's viewing ability has also undergone profound changes. We can see from Figure 9 that if the mental health quality is lacking, it is bound to be difficult for the athletes to better adapt to the competition rhythm. Practice shows that the importance of athletes' psychological quality in their comprehensive quality is becoming more and more prominent, and it plays a pivotal role in competitive performance. Therefore, improving the level of psychological quality education and strengthening the psychological quality of athletes can better face failure and success, joy and tears.

Competitive sports under the commodity economy and social environment presents many new features, of which economy and utility are typical representatives. Under the impact of economic interests, some athletes pay too much attention to their own performance, put too much energy into utilitarianism, and even distort their self-values and outlook on life. Development requirements, unable to realize self-worth. Therefore, in the economic society, it is necessary to make athletes face fame and fortune more correctly and adapt to the needs of social development through systematic psychological education.

(11) $$\begin{array}{c} \chi^{2}=\sum \frac{{\left(f_{o}-f_{e}\right)}^{2}}{f_{e}}. \end{array}$$

Among them, *f*_o_ represents the observed frequency; *f*_*e*_ represents the expected frequency.

Today's sports competition is no longer a purely physical competition, but a comprehensive competition in psychology, training, economy, technology, anti-stress and other aspects. The traditional one simply relies on infinitely extending training time and improving training intensity The method is out of date. At the same time, the age requirements for training and participating in competitive athletes tend to decrease, and their psychological development is relatively lagging behind. All of the above conditions put forward higher requirements for the overall quality of the coaches.

(12) $$\begin{array}{c} I\left(X,Y\right)=\sum_{x\in X}\sum_{y\in Y}P\left(x,y\right)\log\frac{p\left(x,y\right)}{p\left(x\right)p\left(y\right)}. \end{array}$$

Among them, *p (x, y)* represents the joint distribution of two random variables *(X, Y)* of the observed frequency; *p (x)* represents the marginal distribution; *p (y)* represents the marginal distribution.

## 6. Application of BP Neural Network in Intelligent Analysis

**Figure 10:**
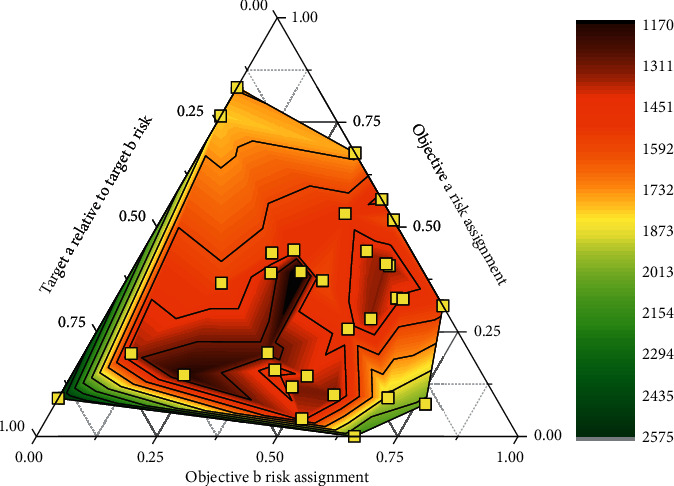
Risk value comparison of two targets.

First, the wavelet transform is used to denoise the athletes' psychological monitoring data, and the denoised data is used as the input of the *BP* neural network. The number of nodes in the input layer depends on the dimension of the data source degree, such as the athlete's mental fitness index plus gender, age, height, and weight. The number of output layer nodes is 3, which are poor, medium, and excellent. The number of hidden layer nodes affects the learning time and training effect. We can see from Figure 10 that the formula is used to determine the number of nodes. Finally, make sure the number of nodes is 6. The network performance is relatively stable. At the same time, the threshold and weight in the network structure are used as the objective function, and the error value between the expected output and the predicted output is used as the fitness function. The optimal solution is obtained by continuous optimization calculation.

In this paper, wavelet analysis is used to denoise, to keep the wavelet coefficients of the effective signal to the greatest extent, and remove the wavelet coefficients of noise. At the same time, in the process of denoising, the smoothness and similarity of the original signal should be kept as far as possible, indicating that the variance estimation of the signal before and after denoising is the minimum value in the worst case. Wavelet analysis has strong denoising ability. The steps of the wavelet analysis method are to select the appropriate wavelet basis function, to decompose the original sample by wavelet, and to obtain the wavelet coefficients of each layer. The wavelet coefficients are processed by the soft threshold method to minimize the maximum mean square error of the estimated signal. The Internet of Things in sports shows the potential to collect and analyze data to improve the performance of spaces, things and athletes. Driven by the competitive nature of sport, iot has the potential to fine-tune its ability to take sports mental health monitoring to a new level.

## 7. Conclusion

For competitive sports, the ultimate goal of sports training is to create excellent sports performance, and the psychological quality of athletes is the most basic factor to improve competitive ability. After training with the training set, the *BP* network and the *PSO-BP* network are tested respectively using the data in the literature, which can accurately reflect the efficiency and accuracy of the *BP* neural network after particle swarm optimization. Aiming at the popularization of smart devices and the needs of athletes' training monitoring, a deep learning-based athlete's physique assessment algorithm was designed. The advantage of IoT technology is that the athletes can remove faults in time and reduce the occurrence of safety accidents in competitive sports. The algorithm scores different athletes by selecting factors such as vertical jumping, high-speed leg raising, sitting and stretching, height, chest circumference, body fat percentage, and uses particle swarm optimization algorithm to optimize the *BP* algorithm to establish athlete psychology. The evaluation model of the situation comprehensively reflects the physical and mental quality of athletes. Through simulation experiments, it is concluded that the optimized *BP* network has high efficiency and accuracy. It is obviously superior to the traditional *BP* network in the evaluation and prediction of athletes' psychological quality.

## Data Availability

The data used to support the findings of this study are available from the corresponding author upon request.

## References

[1] Grassi A., Vascellari A., Vascellari A. (2016). Return to sport after ACL reconstruction: a survey between the Italian society of knee, arthroscopy, sport, cartilage and orthopaedic technologies (SIGASCOT) members. *European Journal of Orthopaedic Surgery and Traumatology*.

[2] Barber-Westin S. D., Noyes F. R. (2011). Factors used to determine return to unrestricted sports activities after anterior cruciate ligament reconstruction. *Arthroscopy: The Journal of Arthroscopic & Related Surgery*.

[3] van Yperen D. T., Reijman M., van Es E. M., Bierma-Zeinstra S. M. A., Meuffels D. E. (2018). Twenty-Year follow-up study comparing operative versus n treatment of anterior cruciate ligament ruptures in high-level athletes. *The American Journal of Sports Medicine*.

[4] Rosa B. B., Asperto A. M., Helito C. P., Demange M. K., Fernandes T. L., Hernandez A. J. (2014). Epidemiology of sports injuries on collegiate athletes at a single center[J]. *Acta Ortopédica Brasileira*.

[5] Allhoff F., Henschke A. (2018). The internet of things: foundational ethical issues. *Internet of Things*.

[6] Makhni E. C., Crump E. K., Steinhaus M. E. (2016). Quality and variability of online available physical therapy protocols from academic orthopaedic surgery programs for anterior cruciate ligament reconstruction. *Arthroscopy: The Journal of Arthroscopic & Related Surgery*.

[7] Arslan G. (2021). Loneliness, college belongingness, subjective vitality, and psychological adjustment during coronavirus pandemic: development of the College Belongingness Questionnaire. *Journal of Positive School Psychology*.

[8] Buckthorpe M., Frizziero A., Roi G. S. (2019). Update on functional recovery process for the injured athlete: return to sport continuum redefined. *British Journal of Sports Medicine*.

[9] Britt R. K., Collins W. B., Wilson K., Linnemeier G., Englebert A. M. (2017). eHealth literacy and health behaviors affecting modern college students: a pilot study of issues identified by the American college health association. *Journal of Medical Internet Research*.

[10] Lei L., Tan Y., Zheng K., Liu S., Zhang K., Shen X. (2020). Deep reinforcement learning for autonomous internet of things: model, applications and challenges. *IEEE Communications Surveys & Tutorials*.

[11] Harrer M., Adam S. H., Fleischmann R. J. (2018). Effectiveness of an internet-and app-based intervention for college students with elevated stress: randomized controlled trial[J]. *Journal of Medical Internet Research*.

[12] Rodrigues J. J. P. C., De Rezende Segundo D. B., Junqueira H. A. (2018). Enabling technologies for the internet of health things. *Ieee Access*.

[13] Lattie E. G., Adkins E. C., Winquist N., Stiles-Shields C., Wafford Q. E., Graham A. K. (2019). Digital mental health interventions for depression, anxiety, and enhancement of psychological well-being among college students: systematic review. *Journal of Medical Internet Research*.

[14] Kim J., Choue R., Lim H. (2016). Differences of sebehavior, diet quality and quality of life in south Korean women according to their weight status. *Clinical nutrition research*.

[15] Jost J. T. (2017). The marketplace of ideology: “Elective affinities” in political psychology and their implications for consumer behavior. *Journal of Consumer Psychology*.

[16] Lee D. Y., Karim S. A., Chang H. C. (2008). Return to sports after anterior cruciate ligament reconstruction - a review of patients with minimum 5-year follow-up. *Annals Academy of Medicine Singapore*.

[17] Grimmelikhuijsen S., Jilke S., Olsen A. L., Tummers L. (2017). Behavioral public administration: combining insights from public administration and psychology. *Public Administration Review*.

[18] Zilio D. (2016). On the autonomy of psychology from neuroscience: a case study of skinner’s radical behaviorism and behavior analysis. *Review of General Psychology*.

[19] Wood W., Rünger D. (2016). Psychology of h. *Annual Review of Psychology*.

[20] Sheeran P., Bosch J. A., Crombez G. (2016). Implicit processes in health psychology: d. *Health Psychology*.

[21] Childs K. E., Kincaid D., George H. P., Gage N. A. (2016). The relationship between school-wide implementation of positive behavior intervention and supports and student discipline outcomes. *Journal of Positive Behavior Interventions*.

[22] Litwiller B., Snyder L. A., Taylor W. D., Steele L. M. (2017). The relationship between sleep and work: a meta-analysis. *Journal of Applied Psychology*.

[23] Howard J. S., Lembach M. L., Metzler A. V., Johnson D. L. (2016). Rates and determinants of return to play after anterior cruciate ligament reconstruction in national collegiate athletic association division I soccer athletes. *The American Journal of Sports Medicine*.

